# Age and Gender Are Associated with the Component of Psychosocial Impact of Dental Aesthetics Questionnaire in Young People: A Cross-Sectional Study

**DOI:** 10.3390/children9040496

**Published:** 2022-04-02

**Authors:** Wan Nurazreena Wan Hassan, Mohd Zambri Mohamed Makhbul, Siti Adibah Othman

**Affiliations:** 1 Department of Paediatric Dentistry and Orthodontics, Faculty of Dentistry, Universiti Malaya, Kuala Lumpur 50603, Malaysia; sitiadibah@um.edu.my; 2 Ministry of Health Malaysia, Orthodontic Unit, Klinik Pergigian Cahaya Suria, Pudu Sentral, Kuala Lumpur 55100, Malaysia; dr.zambri@moh.gov.my

**Keywords:** quality of life, adolescents, young adults, malocclusion

## Abstract

(1) The aim of the study was to investigate the association between age, gender, and the component of psychosocial impact of dental aesthetics (PIDAQ) in Malaysian young people. (2) Cross-sectional data on the PIDAQ (comprised of dental self-confidence, social impact, psychological impact, and aesthetic concern variables) of Malaysian youth (n = 1425) recruited through multi-stage sampling were analyzed for mediation and moderated mediation analyses using the PROCESS macro on SPSS software. (3) Participants (mean age 16.0 ± 2.8) represented 54.8% of girls and 45.2% of boys. In the mediation model, psychological impact and aesthetic concern completely mediated the effects of social impact on dental self-confidence. In the moderated mediation model, social impact directly influenced dental self-confidence amongst participants at one standard deviation below the sample mean age and among boys. However, psychological impact completely mediated the influence of social impact on dental self-confidence amongst participants at the sample mean age and at one standard deviation above the sample mean ages, and among girls. Neither age nor gender moderated the effect of aesthetic concern on dental self-confidence. (4) Age and gender moderated the influence of social impact and psychological impact on dental self-confidence.

## 1. Introduction

Dental self-confidence is a positive sense of well-being related to one’s own dental alignment [1]. Dental appearance affects perceptions of social attractiveness. Adolescents perceived those having ideal smiles to be more athletic and more popular, and to have better leadership abilities than those with non-ideal smiles [2]. Unattractive children become targets for bullying and teasing [3] because they are perceived to lack desirable characteristics assumed to be possessed by attractive individuals [4]. These victims are more likely to be shyer in public as well reticent and reluctant to reveal their teeth.

A smile is a facial expression conveying being pleased, kind, or amused, typically with the corners of the mouth turned up and exposing the front teeth [5]. Individuals who are highly satisfied with their smile like to show their teeth while smiling and like to see their teeth in their reflections or images, exhibiting self-confidence [6]. Smiling can have emotionally and physiologically positive effects. Smiling can reduce the heart rate and stress levels [7]. Smiling positively influences facial [8] and physical attractiveness [9]. Smiling, even when faked, alters the mind to presume facial and bodily expressions in positive emotions [10]. Contrastingly, treatments to remove laughter lines increased depression [11]. A low dental self-confidence manifests in a hesitation to smile and, consequently, the one with low dental self-confidence loses out from the benefits of smiling.

Although the severity of malocclusion is a common reason for orthodontic treatment [12,13,14], one of the motivating factors for seeking treatment is to improve the confidence to smile [15,16]. Since hiding teeth while smiling reflects embarrassment, treatments should focus on restoring dental aesthetics sufficiently for patients to feel confident about smiling [17]. This may include orthodontics because malocclusion has been statistically associated with confidence in smiling [18].

The psychosocial impact of dental aesthetics questionnaire (PIDAQ) measures the extent to which the dental arrangement and smile aesthetics affect an individual’s psychological and social well-being. It is factored into four domains, including three negative domains comprising the social impact (SI) domain that measures anxiety levels about other people’s reaction when one exposes one’s teeth, psychological (PI) domain that measures negative emotions about dental appearance, and aesthetic concern (AC) domain that measures displeasure with the image of one’s teeth in different conditions. The fourth dental self-confidence (DSC) domain measures the impact of dental aesthetics on a positive self-concept [19]. Developing knowledge on the causal processes linking the different aspects of well-being can facilitate clinicians to have a better understanding of how the psychosocial variables affect patients with malocclusion and potentially guide them to manage orthodontic health service more effectively [20].

Much attention has been devoted to the social origins of thought besides the mechanisms through which social factors exert their influence on cognitive functioning and behavioral response [21]. Evidence on the extent to which social influences impact an individual’s dental self-confidence is inconclusive. Although severe malocclusion has been found to affect social and emotional well-being [22], children with overjet did not necessarily have a lower self-concept [23]. Furthermore, social impact is expected to reduce with age [24], yet no evidence has supported any improvement in dental self-confidence with maturity. So, how does social impact affect dental self-confidence? Indeed, the underlying mechanism by which the dental self-confidence variable is affected by the psychosocial variables warrants exploration.

The aim of the study was to investigate the association between age, gender, and the component of PIDAQ in Malaysian young people. It evaluated a moderated mediation relationship to assess the influence of social impact on dental self-confidence among young people. Psychological impact and aesthetic concern were proposed as mediators while age and gender were set as moderators in this relationship model. The null hypotheses were: DSC in PIDAQ is not associated with other components (SI, PI, and AC) and age and gender are not associated with SI, PI, and AC.

The outcome of the study may help clinicians identify characteristics of those patients who may be vulnerable to having low confidence in smiling.

## 2. Materials and Methods

### 2.1. Study Design

This was a cross-sectional study. The research question was examined from biserial epidemiological studies on Malaysian youth [25,26] conducted between May 2016 and August 2018.

### 2.2. Sample

“Young people” were defined as adolescents and young people from 10 to 24 years old as categorized by the World Health Organization and it can be extended to 30 or 40 years old based on a survey poll of the Society for Adolescent Health and Medicine’s International Members [27]. Sample size calculation was not done as analysis was performed on previously collected data. Participants were recruited through multi-stage sampling from five secondary schools and four tertiary institutions in Malaysia. In brief, the country was divided into five regions. For the secondary school sampling, one state per region was randomly selected, followed by random selection of one school per state from a list of secondary schools within the state. For the tertiary institution sampling, one region was randomly selected, followed by random selection of a state within the region. One institution each from the four types of tertiary institutions (university, matriculation, polytechnic, and community college) were randomly selected from a list of tertiary institutions within the selected state. Exclusion criteria barred participants with orthodontic experience, craniofacial deformities, or learning and reading difficulties. Participants who did not disclose their demographic information and had more than 20% of items per domain missing were removed. Final data were gathered on 1425 subjects.

Fifteen subjects had one missing data point while one subject had a missing data point from each of the DSC, SI, and PI domains. Therefore, their missing scores were imputed using the personal mean score method, which was calculated as the mean items of the scale [25].

### 2.3. Measures

In all study samples, the validated 22-item Malaysian PIDAQ [28,29] was self-administered either inside or outside the classroom, school hall, or canteen in printed format or in online format using participants’ own devices. Participants rated each item using a five-point Likert scale from 0 (“not at all”) to 4 (“very strongly”). The internal consistency of the pooled data for each variable was satisfactory with Cronbach α scores of 0.87 for DSC (6 items), 0.86 for PI (6 items), 0.89 for SI (8 items), and 0.69 for AC (2 items) domains.

Participants also rated their own dental attractiveness using the aesthetic component of the index of orthodontic treatment need (IOTN-AC) [30]. They rated the severity of their malocclusion based on a 10-point photographic scale from the most beautiful dental appearance (score 1) to the least beautiful dental appearance (score 10). Their self-perceived malocclusion was categorized as none (score 1 and 2), mild (score 3 and 4), moderate (score 5 to 7), and severe (score 8 to 10).

The demographic variables analyzed included gender (male and female), age, ethnicity (Malay, Chinese, Indian, and other), and self-perceived malocclusion (none, mild, moderate, and severe).

### 2.4. Statistical Procedures

SPSS v26.0 was used for statistical analyses. Descriptive statistics, independent t-test, and one-way analysis of variance (ANOVA) were used to describe and compare the demographic data and distribution of DSC. Correlations between the study variables (DSC, SI, PI, AC, age, and gender) were examined using Spearman correlation coefficients. A correlation of less than 0.10 was considered negligible, up to 0.39 as weak, up to 0.69 as moderate, up to 0.89 as strong, and above 0.90 as very strong [31].

**Figure 1:**
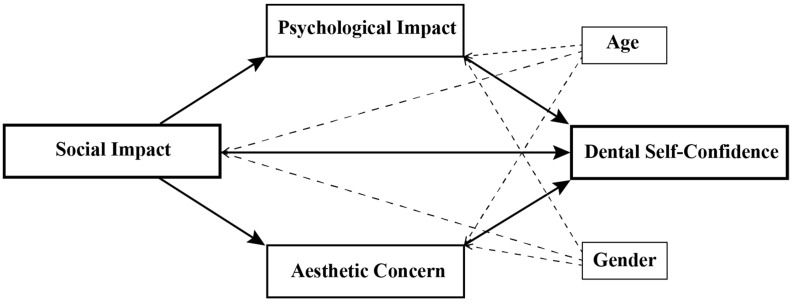
Hypothesis model.

The mediation and moderation analyses were conducted with the demographic variables treated as covariates in the regression equations when relevant. A hypothesis was presented when modelling the independent variable, SI, to predict the dependent variable, DSC. Two independent variables, namely PI and AC, were modelled as mediators in this relationship as a mechanism through which SI may indirectly affect the DSC. In addition, age and gender were modelled as moderators by interacting with SI, PI, and AC on DSC, thereby affecting the magnitude of the effect of the independent variables on the dependent variable (Figure 1).

Mediation, followed by moderated mediation analyses, was performed using the PROCESS macro on SPSS [32]. The analyses used 5000 bootstrap samples at the 95% confidence interval. To assess for a moderation effect, conditional indirect effects were assessed at the mean (at $\bar{X}$) at one standard deviation below the mean age (at $\bar{X}$ − 1 SD) and at one SD above the mean (at $\bar{X}$ + 1 SD) values when age was the moderator variable of interest, as well as between male and female when gender was the moderator variable of interest.

## 3. Results

### 3.1. Demographics and Preliminary Analyses

Table 1 shows the participants’ characteristics and distribution of the DSC scores. The participants comprised 644 boys (45.2%) and 781 girls (54.8%). Boys had a significantly higher DSC than girls (*p* < 0.05). The ethnic composition was Malays (68.6%), Chinese (16.8%), Indians (6.9%), and other (7.6%). Significant differences were noted in the mean DSC between the ethnicities (*p* < 0.05). The mean age of the study population was 16.0 ± 2.8 years. The population age range was 12 to 30 years old. The majority of participants did not have self-perceived malocclusion (55.2%), followed by those who felt they had mild (36.5%), moderate (5.5%), and severe malocclusions (2.8%). DSC scores between participants in these categories were significantly different (*p* < 0.05).

Table 2, regarding the Spearman correlation between the variables of interest, shows that DSC, SI, PI, and AC had moderate to strong correlations with each other (*p* < 0.05). The mean domain scores were 12.8 ± 5.3 for DSC, 9.8 ± 6.8 for SI, 10.3 ± 5.4 for PI, and 2.6 ± 1.9 for AC. All highest and lowest possible scores for these domains were rated by the participants. The correlations of age with these domains were negligible (r ≤ 0.1; *p* < 0.05) or non-significant (*p* > 0.05). The point-biserial correlation also showed that gender had negligible (r ≤ 0.1; *p* < 0.05) correlations with the domains of interest.

### 3.2. Mediation Analysis

Table 3, regarding the mediation analysis, shows that SI positively affected PI and AC (*p* < 0.001) but did not significantly influence DSC (*p* > 0.05). PI and AC had negative effects on DSC (*p* < 0.001). The bootstrapped 95% confidence interval values confirmed significant indirect effects where PI and AC completely mediated the relationship between SI and DSC.

### 3.3. Moderated Mediation Analysis

Table 4, regarding the moderated mediation analysis with age as a moderator, shows that, similarly, PI and AC were found as mediators in the relationship. Furthermore, significant interactions between age and SI, as well as between age and PI on DSC (*p* = 0.014 and <0.001, respectively), were noted, supporting the moderation effects of age. Conversely, age did not moderate the effect of AC on DSC because the interaction between age and AC on DSC was not significant (*p* > 0.05).

**Figure 2:**
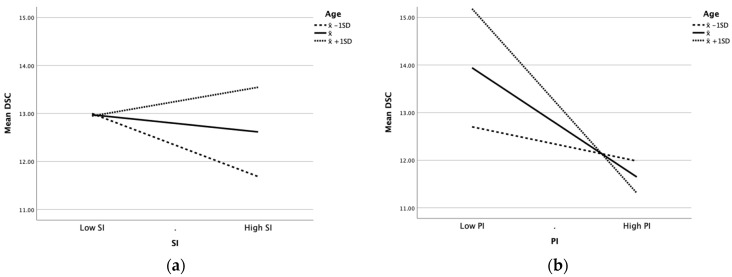
Moderating effect of age in the relationship between (**a**) social impact (SI) on dental self-confidence (DSC) and (**b**) psychological impact (PI) on DSC.

Figure 2a illustrates the moderating effect of age in the relationship between SI and DSC while Table 4 shows the conditional direct effects analysis at the mean age and at one SD above and below it. The younger participants (at $\bar{X}$ − 1 SD) showed a steeper negatively correlated line when compared to the average and older participants, with a conditional effect of SI that was statistically significant (effect = −0.10; *p* = 0.011; 95% CI = −0.17 to −0.02). The average (at $\bar{X}$; *p* = 0.339) and older participants (at $\bar{X}$ + 1 SD; *p* = 0.285) showed insignificant correlations between SI and DSC.

Figure 2b illustrates the moderating effect of age on the effect of PI on DSC. The younger participants (at $\bar{X}$ − 1 SD) showed a relatively plateaued line with a conditional effect of PI that was not significant (*p* = 0.165). The average participants (at $\bar{X}$) had a steeper negatively correlated line (effect = −0.21; *p* < 0.001; 95% CI = −0.28 to −0.14) while the older population (at $\bar{X}$ + 1 SD) showed the steepest negatively correlated line (effect = −0.34; *p* < 0.001; 95% CI = −0.47 to −0.25). Thus, increasing age amplified the negative effects of PI on DSC.

The effect of age in the relationship between SI on DSC with PI as the mediator was also examined by a conditional indirect effects analysis using the bootstrap method (Table 4). With increasing age, the conditional indirect effect of SI on DSC became more negative through PI. The effect was insignificant for the younger participants (at $\bar{X}$ − 1 SD; 95% CI includes 0). However, the effect became more pronounced with increasing age. The influence of SI amongst the participants at the mean age (at $\bar{X}$) and older (at $\bar{X}$ + 1 SD) was significantly associated with DSC via PI (95% CI excludes 0).

Table 5, regarding the moderated mediation analysis when treating gender as a moderator, similarly supports AC as a mediator of the relationship between SI and DSC. Nonetheless, differences were noted in the analysis: SI directly affected DSC (*p* < 0.001) while PI initially did not mediate this relationship (*p* = 0.321). Gender was interpreted to moderate the effects SI and PI on DSC because its interactions with SI, as well as with PI, were significant (*p* = 0.001 and 0.006, respectively). In contrast, age did not moderate the relationship between AC and DSC because its interaction with AC was insignificant (*p* = 0.693).

**Figure 3:**
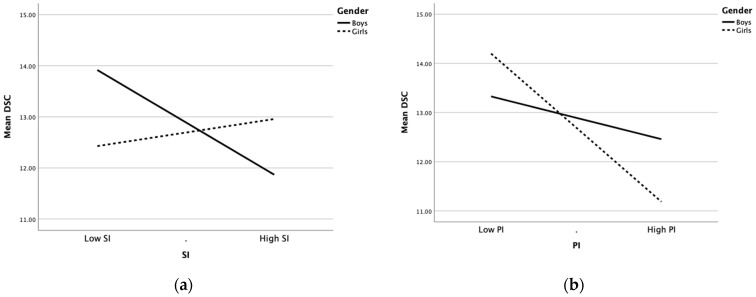
Moderating effect of gender in the relationship between (**a**) social impact (SI) on dental self-confidence (DSC) and (**b**) psychological impact (PI) on DSC.

Figure 3a illustrates the moderating effect of gender in the effect of SI on DSC while Table 5 shows the conditional direct effects analysis between boys and girls. Boys showed a steeper negatively correlated line, with a conditional effect of SI that was statistically significant (effect = −0.15; *p* = 0.001; 95% CI = −0.24 to −0.07). The correlation line for girls was insignificant (*p* = 0.272).

Figure 3b illustrates the moderating effect of gender in the influence of PI on DSC. Boys showed a gentle negatively correlated line, with a conditional effect of PI that was not significant (*p* = 0.144), while girls had a sharp negatively correlated line (effect = −0.28; *p* < 0.001; 95% CI = −0.37 to −0.19). Thus, female gender intensified the negative effects of PI on DSC.

The effect of gender in the relationship of SI on DSC with PI as the mediator was also examined by a conditional indirect effects analysis using the bootstrap method (Table 5). The conditional indirect effect of SI on DSC was not significant for boys (95% CI includes 0). However, in girls, SI was significantly associated with DSC via PI (effect = −0.17; 95% CI = −0.28 to −0.11).

## 4. Discussion

**Figure 4:**
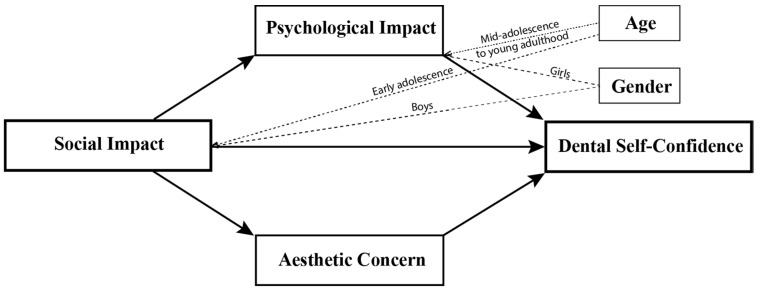
Final model showing that the relationship between social impact and dental self-confidence was mediated by psychological impact and aesthetic concern, and moderated by age and gender.

The present study found that dental self-confidence is influenced by psychosocial factors. Initially, the mediation analysis showed that the effect of social impact on dental self-confidence was completely mediated by psychological impact and aesthetic concern. When age and gender were considered, the moderated mediation results showed that social impact affected dental self-confidence through more complex mechanisms under different conditions: (1) directly amongst early adolescents and boys; (2) indirectly through psychological impact amongst older participants and girls; and (3) indirectly through the aesthetic concern variable. Figure 4 depicts the final moderated mediation model.

This relationship model can be applied to the group of general Malaysian young people since the analysis comprised data of a large sample (n = 1425) recruited by a multi-stage sampling technique to minimize bias. The study included young people aged 12 to 30 years old. This age range is representative of the common orthodontic treatment-seekers. However, since our participants did not comprise the upper and lower spectrum of Malaysian young people, we should pay attention to the generalizability.

It should be kept in mind that the social cognitive theory assumes a reciprocal causation such that personal, behavioral, and environmental factors operate as interacting determinants that influence each other bidirectionally [21]. Certainly, the current study was not claiming a unidirectional relationship but was trying to understand how SI affected dental self-confidence. We only investigated the direction from SI, PI, and AC to DSC. However, these are the components of PIDAQ. We should consider the inverse directions.

Malocclusion is, while perhaps visible, usually asymptomatic, causing neither pain nor discomfort. Thus, the motivation for seeking orthodontic treatment is mainly related to the impacts of dental aesthetics on personal well-being. Patient satisfaction with treatment outcome is essential for them to flaunt their teeth confidently when smiling. It is concerning, then, when more than three-quarters of young people (78%) report of impacts on their dental self-confidence [25]. Therefore, we explored factors that may perhaps modify their dental self-confidence. Efforts to address this problem could facilitate improvement in their ability to interact socially with confidence.

### 4.1. Direct Influence of Social Impact on Dental Self-Confidence in Boys and Young Adolescents

The initial mediation analysis did not find a direct role for social impact on dental self-confidence. However, upon examining the moderated mediation outcome, dental self-confidence was directly influenced by social impact in young adolescents and boys, supporting age and gender as moderators in this relationship. The finding suggested that boys with high social impacts were more likely to have lower dental self-confidence than boys with low social impacts. Social impacts may arise from bullying and media exposure. Boys and young schoolchildren are frequently bullied because of their teeth [33]. Moreover, boys in urban areas are also exposed to a metrosexual lifestyle with demands for personal attractiveness. Males in urban areas have similar impacts related to dental aesthetics as urban females, while males in suburban and rural areas have significantly lower impacts than females in suburban and rural areas [26].

Our study demonstrated that the direct effect of social impact to lower dental self-confidence only occurred in younger participants. With age, social impact did not directly affect their dental self-confidence. Young children are vulnerable to social influences because their comprehension and attitudes are guided by external standards. It is probable that the bullying and teasing of children with malocclusion [3] caused them to conform under peer pressure influence [34]. The child loses trust in their own physical attributes under the mockery over their dental arrangement. As they become wiser with age, they are able to self-reflect and self-regulate to shape their behavior [21], and become less sensitive to external standards. Other factors such as supportive parental upbringing strategies may be able to mold children’s internal locus of control [35], hence they become empowered to take charge over events in their life. Inevitably, maturity may reduce conformity [36] and simultaneously facilitate a reduction in social impact.

### 4.2. Indirect Influence of Social Impact on Dental Self-Confidence via Psychological Impact in Older Participants and Girls

The mediation analysis showed psychological impact as a mediator. The subsequent moderated mediation analysis found that the effects of psychological impact on dental self-confidence were significant in girls and in the older participants, confirming that age and gender again acted as moderators. Social ties can play a role in one’s psychological well-being [37]. Adolescents commonly respond to interpersonal social stressors involving peers. The consequence of being rejected or neglected by peers can lead to psychological stress [38]. The experiences from being teased about one’s teeth during childhood may linger in one’s memory and gradually erode self-confidence [39]. Evidence supported the interpretation that dissatisfaction over dental appearance affects dental self-confidence [40,41]. Self-esteem of undergraduate students, particularly girls, was negatively correlated with teasing experienced during childhood [42].

This study further revealed that social impact, which caused a psychological impact in girls, continued to make them dwell on their dental appearance, downgrading their dental self-confidence. This was not unexpected as females are generally prone to psychological impacts [24]. Insecurity regarding one’s appearance, shaped by media ideals, is driven by competitiveness to appear more attractive than others [43], leading them to find themselves wanting. Based on our findings, it seemed that if girls can be shaped to reduce the psychological impact, they would have much higher dental self-confidence. Therefore, it is suggested that their psychological well-being be dealt with by means of supportive strategies [35] so they are not easily affected by media and peer influences for improved self-confidence.

### 4.3. Indirect Influence of Social Impact on Dental Self-Confidence via Aesthetic Concern

Our study also found that social impact influenced dental self-confidence via aesthetic concern in a relationship that is moderated by neither age nor gender. Adolescence is an age where physical self-consciousness becomes acute and popular standards of beauty begin to have an impact [44]. Their self-awareness is heightened as a consequence of repeated reminders by thoughts of being judged [39]. Self-consciousness about one’s physical appearance is commonly triggered by comments or teasing, and the self-consciousness caused by specific facial features is maintained by self-scrutiny in mirrors or when comparing oneself to real or enhanced images [45]. Those with a noticeable malocclusion desire physical attractiveness [46], in which dental alignment is an important element [47]. These adolescents may not yet have self-confidence because they have not reached a level of maturity to trust in their own abilities, qualities, and judgement [44].

### 4.4. Study Limitations

The lowest age was 12 years old because the psychometric validity of the PIDAQ for subjects below this age has not been confirmed. Population sampling was challenging and focused on subjects who attended learning institutions and was generally comprised of young people. Therefore, application of the outcome of this study to the middle-aged or elderly may not be relevant. Interpretation relating to changes with age should also be taken with caution due to cross-sectional sampling. Further longitudinal research is recommended to validate the study conclusions.

### 4.5. Recommendations from the Study Outcome

The study implies that the approach for managing orthodontic patients be tailored to their personal conditions.

First, the mechanism for the effect of social impact on dental self-confidence is different between boys and girls. In boys, dental self-confidence was directly determined by the degree of social impact experienced, while in girls, dental self-confidence was indirectly affected through psychological impact. Thus, counselling is recommended to be incorporated in the management of psychological distress in impacted patients by training the dental team [48] to identify patients, particularly girls, with low self-confidence. This may provide a more holistic approach in treating orthodontic patients for improved well-being.

Second, the mechanism for the effect of social impact on dental self-confidence changes with age. Our study found that social impact, which had induced a psychological impact, lingered in memory. Despite the patient’s advancing years, psychological impact continued to affect dental self-confidence. The moderating effect of age showed that older individuals with low psychological impact have higher dental self-confidence than younger individuals of the same level of psychological impact. Therefore, in subjects with low psychological impact, improvement in their dental self-confidence was facilitated by maturity. If the intention in seeking orthodontics was to improve dental self-confidence, the healthcare policy for limiting subsidized treatment to adolescents of up to 18 years [49] is reasonable since dental self-confidence becomes stronger with maturity, especially in those who experience low psychological impact.

On the contrary, older individuals with increasing psychological impact had greatly reduced, on a greater level, dental self-confidence than younger adolescents of the same level of psychological impact. Hence, if the aspiration for improved dental self-confidence is the driving force for seeking treatment, national healthcare provisions should not discriminate against individuals who sought for treatment later such that they missed the cut-off of 18 years of age for enrolling themselves in subsidized treatment. Thus, it is recommended that the age limit for subsidized treatment be extended, particularly for older individuals with high psychological impact. In a state where limited funding restricts the offering of treatment to a larger population, policy-makers can consider a targeted approach using impact-related needs for prioritizing treatment [50]. Such an evidence-based approach may potentially provide treatment without agism to those impacted by their malocclusion.

## 5. Conclusions

Age and gender are associated with the component of PIDAQ in Young People.

1. SI was directly and indirectly associated with DSC. PI and AC were directly associated with DSC.
2. Age and gender acted as moderators to modify the strength of the association. In girls and in older adolescents as well as young adults, the effect of SI was more deeply rooted, occurring through the PI, which, in turn, influenced DSC.

## Figures and Tables

**Table 1:** Participants’ characteristics and distribution of dental self-confidence (DSC) scores.

Variable	Groups	N (%)	DSC Score		Statistics		*p*
			Mean	SD	t	F	
Gender					3.289		0.001 *
	Boys	644 (45.2)	13.3	5.2			
	Girls	781 (54.8)	12.3	5.4			
Ethnicity						7.088	<0.001 *
	Malay	978 (68.6)	13.0	5.0			
	Chinese	240 (16.8)	11.5	6.0			
	Indian	99 (6.9)	14.1	5.9			
	Other	108 (7.6)	12.4	5.5			
Age			16.0	2.8		2.133	0.094
	14 and below	571 (40.1)	12.4	5.2			
	15 to 19	708 (49.7)	13.1	5.3			
	20 to 24	142 (10.0)	12.7	5.7			
	25 to 30	4 (0.3)	12.5	5.4			
Self-perceived malocclusion						86.987	<0.001 *
	None	787 (55.2)	14.5	5.1			
	Mild	520 (36.5)	11.2	4.7			
	Moderate	78 (5.5)	7.9	4.4			
	Severe	40 (2.8)	8.2	4.5			

* *p* < 0.05 and standard deviation (SD).

**Table 2:** Correlations between the variables of interest and their descriptive statistics.

Variables	DSC	SI	PI	AC	Mean	S.D.	Min	Max
DSC: dental self-confidence	1	−0.487 ***	−0.527 ***	−0.582 ***	12.8	5.3	0	24
SI: social impact		1	0.791 ***	0.676 ***	9.8	6.8	0	32
PI: psychological impact			1	0.699 ***	10.3	5.4	0	24
AC: aesthetic concern				1	2.6	1.9	0	8
Age	0.043	0.080 **	0.057 *	0.089 **	16.0	2.8	12	30
Gender ^ø^	−0.087 **	0.071 **	0.109 ***	0.102 ***				

* *p* < 0.05; ** *p* < 0.01; *** *p* < 0.001; and ^ø^ point-biserial correlation and otherwise Spearman.

**Table 3:** Mediation analysis.

Direct Effects							
Variables	B	SE	t	*p*	LLCI	ULCI	
SI → PI	0.60	0.01	45.01	<0.001	0.57	0.62	*
SI → AC	0.19	0.01	33.70	<0.001	0.17	0.20	*
SI → DSC	−0.04	0.03	−1.37	0.171	−0.09	0.02	
PI → DSC	−0.20	0.04	−5.45	<0.001	−0.27	−0.13	*
AC → DSC	−1.05	0.09	−12.06	<0.001	−1.23	−0.88	*
R^2^ (PI): 0.656 (*p* < 0.001); R^2^ (AC): 0.513 (*p* < 0.001); R^2^ (DSC): 0.430 (*p* < 0.001)							
**Indirect Effects** (SI → mediator → DSC)							
**Variables**	**Effect**	**BootSE**			**BootLLCI**	**BootULCI**	
PI	−0.12	0.02			−0.16	−0.07	*
AC	−0.19	0.02			−0.23	−0.16	*
Total	−0.31	0.03			−0.36	−0.26	*

* *p* < 0.05 and/or LLCI to ULCI exclude zero. Legend: social impact (SI), psychological impact (PI), aesthetic concern (AC), dental self-confidence (DSC), and lower limit and upper limit confidence intervals (LLCI and ULCI).

**Table 4:** Moderated mediation analysis when treating age as a mediator.

Direct Effects							
Variables	B	SE	t	*p*	LLCI	ULCI	
SI → PI	0.59	0.01	45.09	<0.001	0.57	0.62	*
SI → AC	0.19	0.01	33.87	<0.001	0.17	0.20	*
SI → DSC	−0.03	0.03	−0.96	0.339	−0.08	0.03	
PI → DSC	−0.21	0.04	−5.88	<0.001	−0.28	−0.14	*
AC → DSC	−1.05	0.09	−12.02	<0.001	−1.22	−0.88	*
SI*Age → DSC	0.02	0.01	2.47	0.014	0.01	0.05	*
PI*Age → DSC	−0.05	0.01	−0.396	<0.001	−0.08	−0.03	*
AC*Age → DSC	−0.03	0.03	−1.02	0.307	−0.09	0.03	
R^2^ (PI): 0.656 (*p* < 0.001); R^2^ (AC): 0.512 (*p* < 0.001); R^2^ (DSC): 0.442 (*p* < 0.001)							
**Conditional Direct Effects at Specific Levels of Age as Moderator**							
SI → DSC	**B**	**SE**	**t**	***p***	**LLCI**	**ULCI**	
1 SD below mean	−0.10	0.04	−2.55	0.011	−0.17	−0.02	*
Mean	−0.03	0.03	−0.96	0.339	−0.08	0.03	
1 SD above mean	0.04	0.04	1.07	0.284	−0.04	0.13	
**Conditional Indirect Effects at Specific Levels of Age as Moderator**							
SI → PI → DSC	**Effect**	**BootSE**			**BootLLCI**	**BootULCI**	
1 SD below mean	−0.04	0.03			−0.10	0.02	
Mean	−0.13	0.02			−0.17	−0.08	*
1 SD above mean	−0.21	0.03			−0.28	−0.15	*

* *p* < 0.05 and/or LLCI to ULCI exclude zero. Legend: social impact (SI), psychological impact (PI), aesthetic concern (AC), dental self-confidence (DSC), and lower limit and upper limit confidence intervals (LLCI and ULCI).

**Table 5:** Moderated mediation analysis when treating gender as a mediator.

Direct Effects							
Variables	B	SE	t	*p*	LLCI	ULCI	
SI → PI	0.60	0.01	45.23	< 0.001	0.57	0.63	*
SI → AC	0.19	0.01	33.94	< 0.001	0.18	0.20	*
SI → DSC	−0.34	0.09	−3.63	< 0.001	−0.52	−0.16	*
PI → DSC	0.12	0.12	0.99	0.321	−0.12	0.35	
AC → DSC	0.95	0.28	−3.35	0.001	−1.50	−0.39	*
SI * Gender → DSC	0.19	0.07	3.39	0.001	0.08	0.30	*
PI * Gender → DSC	−0.20	0.07	−2.76	0.006	−0.34	−0.06	*
AC * Gender → DSC	−0.07	0.17	−0.39	0.693	−0.41	0.27	
R^2^ (PI): 0.652 (*p* < 0.001); R^2^ (AC): 0.510 (*p* < 0.001); R^2^ (DSC): 0.435 (*p* < 0.001)							
**Conditional Direct Effects at Specific Levels of Gender as Moderator**							
SI → DSC	**B**	**SE**	**t**	***p***	**LLCI**	**ULCI**	
Male	−0.15	0.04	−3.48	0.001	−0.24	−0.07	*
Female	0.04	0.04	1.10	0.272	−0.03	0.11	
**Conditional Indirect Effects at Specific Levels of Gender as Moderator**							
SI → PI → DSC	**Effect**	**BootSE**			**BootLLCI**	**BootULCI**	
Male	−0.05	0.04			−0.12	0.02	
Female	−0.17	0.03			−0.28	−0.11	*

* *p* < 0.05 and/or LLCI to ULCI exclude zero. Legend: social impact (SI), psychological impact (PI), aesthetic concern (AC), dental self-confidence (DSC), and lower limit and upper limit confidence intervals (LLCI and ULCI).

## Data Availability

The data presented in this study are available upon reasonable request from the corresponding author.

## References

[1] Klages U., Bruckner A., Zentner A. (2004). Dental Aesthetics, Self-Awareness, and Oral Health-Related Quality of Life in Young Adults. Eur. J. Orthod..

[2] Henson S.T., Lindauer S.J., Gardner W.G., Shroff B., Tufekci E., Best A.M. (2011). Influence of Dental Esthetics on Social Perceptions of Adolescents Judged by Peers. Am. J. Orthod. Dentofac. Orthop..

[3] Tristão S.K.P.C., Magno M.B., Pintor A.V.B., Christovam I.F.O., Ferreira D.M.T.P., Maia L.C., de Souza I.P.R. (2020). Is There a Relationship between Malocclusion and Bullying? A Systematic Review. Prog. Orthod..

[4] Klages U., Zentner A. (2007). Dentofacial Aesthetics and Quality of Life. Semin. Orthod..

[5] Pearsall J., Hanks P. (2003). Oxford Dictionary of English.

[6] Afroz S., Rathi S., Rajput G., Rahman S.A. (2013). Dental Esthetics and Its Impact on Psycho-Social Well-Being and Dental Self Confidence: A Campus Based Survey of North Indian University Students. J. Indian Prosthodont Soc..

[7] Kraft T.L., Pressman S.D. (2012). Grin and Bear It: The Influence of Manipulated Facial Expression on the Stress Response. Psychol. Sci..

[8] Janson G., Branco N.C., Morais J.F., Freitas M.R. (2014). Smile Attractiveness in Patients with Class II Division 1 Subdivision Malocclusions Treated with Different Tooth Extraction Protocols. Eur. J. Orthod..

[9] Lopez Y., Rouzic J., Bertaud V., Pérard M., Clerc J., Vulcain J. (2013). Influence of Teeth on the Smile and Physical Attractiveness. A New Internet Based Assessing Method. Open J. Stomatol..

[10] Marmolejo-Ramos F., Murata A., Sasaki K., Yamada Y., Ikeda A., Hinojosa J.A., Watanabe K., Parzuchowski M., Tirado C., Ospina R. (2020). Your Face and Moves Seem Happier When I Smile. Exp. Psychol..

[11] Lewis M.B. (2018). The Interactions between Botulinum-Toxin-Based Facial Treatments and Embodied Emotions. Sci. Rep..

[12] Miguel J.A., Sales H.X., Quintao C.C., Oliveira B.H., Feu D. (2010). Factors Associated with Orthodontic Treatment Seeking by 12–15-Year-Old Children at a State University-Funded Clinic. J. Orthod..

[13] Feldens C.A., Nakamura E.K., Tessarollo F.R., Closs L.Q. (2015). Desire for Orthodontic Treatment and Associated Factors among Adolescents in Southern Brazil. Angle Orthod..

[14] Marques L.S., Pordeus I.A., Ramos-Jorge M.L., Filogonio C.A., Filogonio C.B., Pereira L.J., Paiva S.M. (2009). Factors Associated with the Desire for Orthodontic Treatment among Brazilian Adolescents and Their Parents. BMC Oral Health.

[15] Pabari S., Moles D.R., Cunningham S.J. (2011). Assessment of Motivation and Psychological Characteristics of Adult Orthodontic Patients. Am. J. Orthod. Dentofac. Orthop..

[16] Bradley E., Shelton A., Hodge T., Morris D., Bekker H., Fletcher S., Barber S. (2020). Patient-Reported Experience and Outcomes from Orthodontic Treatment. J. Orthod..

[17] Samorodnitzky-Naveh G.R., Geiger S.B., Levin L. (2007). Patients’ Satisfaction with Dental Esthetics. J. Am. Dent. Assoc..

[18] Muniz F., Cavalcante D.J., Moreira M.M.S.M., Rodrigues L.K.A., de Oliveira Fernandes C.A., de Almeida P.C., de Sousa Carvalho R. (2017). Association between Confidence in Smiling and Esthetic Characteristics. J. Esthet. Restor. Dent..

[19] Klages U., Erbe C., Sandru S.D., Brullman D., Wehrbein H. (2015). Psychosocial Impact of Dental Aesthetics in Adolescence: Validity and Reliability of a Questionnaire across Age-Groups. Qual. Life Res..

[20] Masood M., Masood Y., Newton T., Lahti S. (2015). Development of a Conceptual Model of Oral Health for Malocclusion Patients. Angle Orthod..

[21] Bandura A. (2001). Social Cognitive Theory of Mass Communication. Media Psychol..

[22] Bittencourt J.M., Martins L.P., Bendo C.B., Vale M.P., Paiva S.M. (2017). Negative Effect of Malocclusion on the Emotional and Social Well-Being of Brazilian Adolescents: A Population-Based Study. Eur. J. Orthod..

[23] Dann C., Phillips C., Broder H.L., Tulloch J.F. (1995). Self-Concept, Class II Malocclusion, and Early Treatment. Angle Orthod..

[24] Spalj S., Novsak A., Bilobrk P., Katic V., Zrinski M.T., Pavlic A. (2016). Mediation and Moderation Effect of the Big Five Personality Traits on the Relationship between Self-Perceived Malocclusion and Psychosocial Impact of Dental Esthetics. Angle Orthod..

[25] Wan Hassan W.N., Yusof Z.Y.M., Yuen S.W., Mohd Tajudin Z., Lokman N., Makhbul M.Z.M. (2019). Prevalence, Extent and Severity of the Psychosocial Impact of Dental Aesthetics among Malaysian Adolescents. Sains. Malays..

[26] Tajudin Z.M., Wan Hassan W.N., Yusof Z.Y.M., Makhbul M.Z.M. (2021). Impacts of Self Perceived Malocclusion on the Oral Health Related Quality of Life of Young Adults. Healthcare.

[27] Walker-Harding L.R., Christie D., Joffe A., Lau J.S., Neinstein L. (2017). Young Adult Health and Well-Being: A Position Statement of the Society for Adolescent Health and Medicine. J. Adolesc. Health.

[28] Wan Hassan W.N., Yusof Z.Y.M., Makhbul M.Z.M., Shahidan S.S., Mohd Ali S.F., Burhanudin R., Gere M.J. (2017). Validation and Reliability of the Malaysian English Version of the Psychosocial Impact of Dental Aesthetics Questionnaire for Adolescents. Health Qual. Life Outcomes.

[29] Wan Hassan W.N., Yusof Z.Y.M., Shahidan S.S., Mohd Ali S.F., Makhbul M.Z.M. (2017). Validation and Reliability of the Translated Malay Version of the Psychosocial Impact of Dental Aesthetics Questionnaire for Adolescents. Health Qual. Life Outcomes.

[30] Grzywacz I. (2003). The Value of the Aesthetic Component of the Index of Orthodontic Treatment Need in the Assessment of Subjective Orthodontic Treatment Need. Eur. J. Orthod..

[31] Schober P., Boer C., Schwarte L.A. (2018). Correlation Coefficients: Appropriate Use and Interpretation. Anesth. Analg..

[32] The Process Macro for Spss, Sas and R Version v4.0. http://www.processmacro.org/download.html.

[33] Alabdulrazaq R.S., Al-Haj Ali S.N. (2020). Parental Reported Bullying among Saudi Schoolchildren: Its Forms, Effect on Academic Abilities, and Associated Sociodemographic, Physical, and Dentofacial Features. Int. J. Pediatr..

[34] Hajnal A., Vonk J., Hill V.Z. (2020). Peer Influence on Conformity and Confidence in a Perceptual Judgment Task. Psihologija.

[35] Ahlin E.M., Lobo Antunes M.J. (2015). Locus of Control Orientation: Parents, Peers, and Place. J. Youth Adolesc..

[36] Vollmer A.L., Read R., Trippas D., Belpaeme T. (2018). Children Conform, Adults Resist: A Robot Group Induced Peer Pressure on Normative Social Conformity. Sci. Robot.

[37] Kawachi I., Berkman L.F. (2001). Social Ties and Mental Health. J. Urban Health.

[38] Taylor S.E., Repetti R.L., Seeman T. (1997). Health Psychology: What Is an Unhealthy Environment and How Does It Get under the Skin?. Annu. Rev. Psychol..

[39] Taghavi Bayat J., Hallberg U., Lindblad F., Huggare J., Mohlin B. (2013). Daily Life Impact of Malocclusion in Swedish Adolescents: A Grounded Theory Study. Acta Odontol. Scand..

[40] Paula D.F., Silva É.T., Campos A.C., Nuñez M.O., Leles C.R. (2011). Effect of Anterior Teeth Display During Smiling on the Self-Perceived Impacts of Malocclusion in Adolescents. Angle Orthod..

[41] de Paula Junior D.F., Santos N.C., da Silva E.T., Nunes M.F., Leles C.R. (2009). Psychosocial Impact of Dental Esthetics on Quality of Life in Adolescents. Angle Orthod..

[42] Gleason J.H., Alexander A.M., Somers C.L. (2000). Later Adolescents’ Reactions to Three Types of Childhood Teasing: Relations with Self-Esteem and Body Image. Soc. Behav. Pers..

[43] Sides-Moore L., Tochkov K. (2011). The Thinner the Better? Competitiveness, Depression and Body Image among College Student Women. Coll. Stud. J..

[44] Pickhardt C.E. (2019). Adolescence and the Desire for Physical Beauty. Psychol. Today.

[45] Bradbury E. (2012). Self-Consciousness: What Is It and How Does It Relate to Cosmetic Procedures?. J. Aesthet. Nurs..

[46] Ruiz C., Conde E., Torres E. (2005). Importance of Facial Physical Attractiveness of Audiovisual Models in Descriptions and Preferences of Children and Adolescents. Percept. Mot. Skills.

[47] Montero J., Gomez Polo C., Rosel E., Barrios R., Albaladejo A., Lopez-Valverde A. (2016). The Role of Personality Traits in Self-Rated Oral Health and Preferences for Different Types of Flawed Smiles. J. Oral Rehabil..

[48] Hoad-Reddick G. (2004). How Relevant Is Counselling in Relation to Dentistry?. Br. Dent. J..

[49] Widström E., Eaton K.A. (2004). Oral Healthcare Systems in the Extended European Union. Oral Health Prev. Dent..

[50] Wan Hassan W.N., Makhbul M.Z.M., Yusof Z.Y.M. (2021). Use of the Sociodental Approach in Estimating Orthodontic Treatment Needs in Adolescent Patients. J. Orofac. Orthop..

